# Protective variant associated with alcohol dependence in a Mexican American cohort

**DOI:** 10.1186/s12881-014-0136-z

**Published:** 2014-12-21

**Authors:** Trina M Norden-Krichmar, Ian R Gizer, Kirk C Wilhelmsen, Nicholas J Schork, Cindy L Ehlers

**Affiliations:** Department of Molecular and Cellular Neuroscience, The Scripps Research Institute, La Jolla, CA 92037 USA; Department of Psychological Sciences, University of Missouri, Columbia, MO 65211 USA; Department of Genetics and Neurology, University of North Carolina, Chapel Hill, NC 27599 USA; Department of Human Biology, J. Craig Venter Institute, La Jolla, CA 92037 USA

**Keywords:** Alcohol dependence, Alcohol dehydrogenase, ADH, Mexican Americans

## Abstract

**Background:**

Mexican Americans, particularly those born in the United States, are at greater risk for alcohol associated morbidity and mortality. The present study sought to investigate whether specific genetic variants may be associated with alcohol use disorder phenotypes in a select population of Mexican American young adults.

**Methods:**

The study evaluated a cohort of 427 (age 18 – 30 years) Mexican American men (n = 171) and women (n = 256). Information on alcohol dependence was obtained through interview using the Semi-Structured Assessment for the Genetics of Alcoholism (SSAGA). For all subjects, DNA was extracted from blood samples, followed by genotyping using an Affymetrix Axiom Exome1A chip.

**Results:**

A protective variant (rs991316) located downstream from the *ADH7* (alcohol dehydrogenase 7) gene showed suggestive significance in association with alcohol dependence symptom counts derived from DSM-III-R and DSM-IV criteria, as well as to clustered alcohol dependence symptoms. Additional linkage analysis suggested that nearby variants in linkage disequilibrium with rs991316 were not responsible for the observed association with the alcohol dependence phenotypes in this study.

**Conclusions:**

*ADH7* has been shown to have a protective role against alcohol dependence in previous studies involving other ethnicities, but has not been reported for Mexican Americans. These results suggest that variants near *ADH7* may play a role in protection from alcohol dependence in this Mexican American cohort.

## Background

Hispanic American subgroups have diversity with respect to racial heritage and their cultures also vary in economic, religious, and psychosocial bases. The importance of specifying subgroups of Hispanics in order to avoid inaccurate generalizations has been emphasized [1,2]. Mexican Americans are the largest subgroup of Hispanic Americans, representing nearly two-thirds of the total US Hispanic population, and as such are an important target population in need of further study. The prevalence rate of past heavy drinking in Mexican Americans was estimated in one report to be 3 times higher than that reported for a non-Hispanic male population [3]. The Los Angeles site of the Epidemiologic Catchment Area Study found that Mexican American men have higher alcohol dependence rates across all age categories compared to White men [4]. However, in a study by Vega et al. [5], higher overall alcohol dependence rates were found only in US-born Mexican Americans. More recent studies, comparing Hispanic national groups in the United States, show that Mexican Americans, together with Puerto Ricans, have the highest rates of binge drinking, driving under the influence of alcohol, alcohol abuse, and dependence [6-8].

The emerging picture of the genetic component to alcohol dependence suggests that the frequencies of risk and protective alleles associated with alcohol dependence may vary between ethnicities. For example, the genes involved in alcohol metabolism represent candidate genes for alcohol use disorders, and thus, have been the focus of research in a number of different ethnic populations [9,10]. The seven alcohol dehydrogenase (*ADH*) genes, *ADH7*, *ADH1C*, *ADH1B*, *ADH1A*, *ADH6*, *ADH4*, and *ADH5*, are located in a single cluster on chromosome 4q21–24 with each gene coding for a unique isozyme. Some examples of variability in response to alcohol based on differences in *ADH* genotype include variants in *ADH1B* (e.g., ADH1B*2) that confer protection against alcohol dependence in East Asians, as well as Europeans, though at a much lower frequency [11]*.* A proportion of individuals of African origin also have a variant in *ADH1B* (ADH1B*3) that has been demonstrated to be protective against alcohol dependence in African Americans [12] and Afro-Caribbeans [13].

Only a few studies have evaluated polymorphisms in alcohol metabolism genes and the risk for alcohol dependence in Mexican American populations. Of the studies conducted, one study found that the ADH1B*1 and ADH1C*2 alleles were both associated with alcohol dependence in Mexican Americans. The authors concluded that Mexican Americans might have a unique pattern of genetic risk that may be in part responsible for the elevated rates of alcohol dependence and alcohol-associated health problems in this population [14-17]. In a second population of Mexican Americans, presence of at least one ADH1B*2 allele was found in 13% of the population, and was associated with protection against alcohol dependence. No significant associations between alcohol dependence and polymorphisms in *ADH1C* were found in this study [18]. Notably, each of these studies was based on a candidate gene approach and did not use a genome-wide corrected alpha level (i.e., p < 1e-8) to correct for type 1 error.

The aims of the present study were to further explore associations between risk and protective variants and alcohol dependence in a select population of Mexican American young adults. DNA and clinical information on 3 alcohol use disorder phenotypes were collected. In order to further expand the search for potential genes associated with alcohol use disorders, the genome was interrogated using an Affymetrix Exome1A array.

## Methods

### Sample ascertainment

To investigate risk and protective factors for alcohol dependence in a select population of Mexican American young adults, we investigated a cohort of 427 (age 18–30) Mexican American men (n = 171) and women (n = 256). Participants were recruited using a commercial mailing list that provided the addresses of individuals with Hispanic surnames in 11 zip codes in San Diego County. The mailed invitation stated that potential participants must be of Mexican American heritage, between the ages of 18 and 30 years, residing in the United States legally, and able to read and write in English. Based on a phone interview, participants were excluded if they were pregnant, were nursing, or currently had a major medical or neurological disorder or head injury. All participants identified as having over 20% Hispanic heritage, with 92% reporting over 50% Hispanic heritage. Information on alcohol dependence was obtained through interview using the Semi-Structured Assessment for the Genetics of Alcoholism (SSAGA) [19], which was used to make lifetime substance use and other psychiatric disorder diagnoses according to DSM-III-R and DSM-IV criteria. Under DSM-III-R guidelines, there are 9 symptom groups in the criteria for alcohol dependence, while there are 7 symptom groups in the DSM-IV guidelines. In both cases, subjects with the presence of symptoms from 3 or more symptom groups are assigned a diagnosis of alcohol dependence. There have been several studies that have evaluated the concurrent diagnostic validity of the SSAGA across alcohol and drug dependencies, major depression, anxiety disorders, and antisocial personality disorder [19,20]. These findings indicate that the SSAGA is a highly reliable and valid instrument for use in studies of psychiatric disorders, including substance dependence. The protocol for the study was approved by the Institutional Review Board (IRB) at The Scripps Research Institute, and written consent was obtained for all participants. Participants were asked to refrain from alcohol and drug usage for 24 hours prior to the testing.

### Sample preparation and genotyping

For all subjects, DNA was extracted from blood samples, followed by genotyping using an Affymetrix Exome1A chip. The DNA samples were prepared and the exome chip genotyping was performed on the Affymetrix Axiom Exome 1A Array according to the Affymetrix Axiom 2.0 Assay Manual Workflow documentation. The Affymetrix Exome 1A chip contains 247,222 markers. Variant quality from the exome chip genotyping was initially assessed according to Affymetrix best practices [21]. Plink version 1.07 [22] was used to calculate Hardy-Weinberg (HWE) p-values on the set of unrelated samples, followed by the removal of 653 variants with an HWE p < 10^−10^.

### Association analysis

PLINK was used to test for genome-wide association for three phenotypic categories: 1) counts of alcohol dependence symptom groups as defined by DSM-III-R guidelines (phenotype value of 0 through 9), 2) counts of alcohol dependence symptom groups as defined by DSM-IV guidelines (phenotype value of 0 through 7), and 3) a dichotomous variable for DSM-IV alcohol dependence where the symptoms were required to cluster within a one year period. PLINK was run with linear regression model parameters and with one million permutations. Gender and age were included as covariates. In order to test if the markers were in high linkage disequilibrium with each other, PLINK was used to LD prune based on variance inflation factor (VIF = 2), and by pairwise correlation (R2 = 0.5). To reduce the effect of extreme outliers in the phenotypic values, we used a winsorization transformation, whereby the extreme values are replaced by certain percentiles rather than discarding the outliers. In particular, the lowest 5% of the numerically sorted phenotype values were replaced by the 5th percentile value, and the highest 5% of the values were set to the 95th percentile value. Custom R code was written to generate winsorized phenotype values at the 5% and 95% cutoffs, which were then used as the phenotype values in PLINK. Manhattan plots were generated using Manhattan R library (Stephen Turner, http://gettinggeneticsdone.blogspot.com/2011/04/annotated-manhattan-plots-and-qq-plots.html), and the Integrative Genomics Viewer (IGV) [23,24]. Annotations of the variants were obtained from the Affymetrix Exome 1A chip description file. Multiple test correction p-value thresholds were calculated for the Affymetrix Exome1A chip using the Genetic Type 1 Error Calculator (GEC) software [25].

### LD analysis

In order to check for linkage disequilibrium across the *ADH* gene cluster, PLINK was utilized to extract a subset of variants for analysis based on the physical position of the *ADH* gene cluster on chromosome 4. In particular, a region of chromosome 4 from genomic location 99,500,000 to 100,450,000 was extracted from the data set. Haploview [26] was used to calculate the LD statistics and visualize the haplotype block structure of the *ADH* gene cluster region.

### Collapsing/Gene-based analysis

Custom python code implemented by Vikas Bansal for previous studies [27,28] was used to perform three rare variant collapsing methods based on techniques of Madsen and Browning [29], Li and Leal [30], and RareCover [31]. A detailed description of these methods can be found in the original articles above, but briefly, these methods involve combining rare variants into a single set which can be tested for differences between the case and control subjects. The implementation of the Li and Leal method [30] collapses multiple rare variants into a single variable for each subject. This variable is tested for association with the phenotype using the Fisher exact test. The implementation of the Madsen and Browning method [29] tests for the differences in the cases and controls, using the number of rare variants and a weight calculated from minor allele frequency of the rare variants. The implementation of the RareCover method [31] is similar to the Li and Leal method, but instead of collapsing all of the rare variants, it creates and examines a subset of rare variants that are most different in frequency between the cases and the controls. The set of 62 rare variant (MAF <= 0.05) markers in the Affymetrix Exome 1A chip that resided in the *ADH* gene region were supplied as the region of variants to collapse.

## Results

### Demographics of the Mexican American population

The demographics for the full sample of individuals (N = 427) that were included in the association analysis are shown in Table 1. The subjects were a mean age of 23.6 (range 18 – 30) years at the time of interview, with 40% of the sample being male and 60% of the sample being female. Participants had a mean of 13.3 years of education (SD = 1.8), and a mean income of $30,000-$49,000. Ethnic background was self-reported by the subjects, by choosing the dominant heritage of each of their great grandparents from a list of possible ethnicities. For each subject, the numbers of great grandparents reported as Mexican, Mexican-American, Chicano, Mexican Indian, Caribbean, Cuban, Puerto Rican, South American, or other Spanish, were tallied, resulting in a percentage between 0 and 100%. From these tallies, 92% of the participants reported at least 50% Hispanic heritage. The mean BMI was 27 (SD = 7, range 17–64). Approximately 29% of the participants (N = 125) were diagnosed with alcohol dependence according to DSM-III-R guidelines, indicating that these subjects had symptoms from 3 or more symptom groups, out of 9 possible symptom groups. Approximately, 20% of the participants (N = 86) were diagnosed with alcohol dependence according to DSM-IV guidelines, indicating that these subjects had symptoms from 3 or more symptom groups, out of 7 possible symptom groups. Approximately 17% of the participants (N = 74) were diagnosed with alcohol dependence when the DSM-IV symptoms were required to cluster within a one-year period.

**Table 1:** **Demographics for Mexican American study participants**

	**Male**	**Female**	**Total**
**Participants**	171 (40%)	256 (60%)	427
**Age: mean(sd), [range]**	23.8 (3.9), [18–30]	23.5 (3.8), [18–30]	23.6 (3.8), [18–30]
**Self-reported MA heritage > = 50%**	90.60%	92.60%	91.60%
**BMI: mean(sd), [range]**	27.6 (6.0) [17.3-52.3]	27.1 (7.4) [16.9-64.4]	27.3 (6.7) [16.9-64.4]
**Education in years: mean(sd), [range]**	13.2 (1.8), [9–17]	13.4 (1.8), [7–18]	13.3 (1.8), [7–18]
**Income: mean(sd), [range]**	4.8 (1.2), [1–9]	4.2 (2.2), [0–9]	4.5 (2.2) [0–9]
**Number of Subjects Affected:**			
**Alcohol Dependence (AlcDep3)**	63 [63/171 = 36%]	62 [62/256 = 24%]	125 (29.2%)
**Alcohol Dependence (AlcDep4)**	48 [48/171 = 28%]	38 [38/256 = 15%]	86 (20.1%)
**Alcohol Dependence (AlcDepClust)**	43 [43/171 = 25%]	31 [31/256 = 12%]	74 (17.3%)

*Abbreviations:* AlcDep3 = alcohol dependence as defined by DSM-III-R guidelines; AlcDep4 = alcohol dependence as defined by DSM-IV guidelines; AlcDep_Cluster = DSM-IV alcohol dependence within 1 year clustering. Affected alcohol dependence status for AlcDep3 and AlcDep4 is assigned to subjects with 3 or more reported alcohol dependence criteria.

### Association analysis

**Figure 1:**
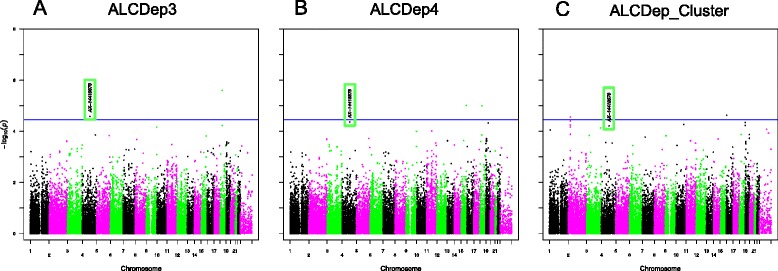
**Manhattan plots for alcohol dependence phenotypes.** Manhattan plots for alcohol dependence phenotypes: **A)** ALCDep3 = alcohol dependence as defined by DSM-III-R guidelines; **B)** ALCDep4 = alcohol dependence as defined by DSM-IV guidelines; **C)** ALCDep_Cluster = DSM-IV alcohol dependence within 1 year clustering. Minor allele frequency cutoff of 0.05 applied to the plot. Suggestive significance line calculated from GEC software. Green rectangle in plots highlights SNP rs991316.

**Figure 2:**
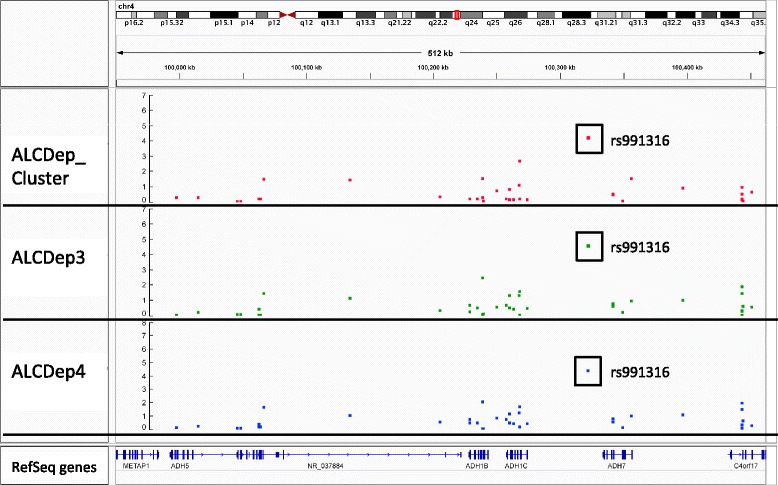
**Manhattan plot of the association results across the** ***ADH*** **gene region.** The *ADH* gene locations are shown in the bottom track of this plot generated by loading the PLINK results into IGV. The black rectangle highlights SNP rs991316, which exhibits a much higher significance than the other SNPs in the *ADH* gene region. No minor allele frequency cutoff was applied. Abbreviations: ALCDep3 = alcohol dependence as defined by DSM-III-R guidelines; ALCDep4 = alcohol dependence as defined by DSM-IV guidelines; ALCDep_Cluster = DSM-IV alcohol dependence within 1 year clustering.

Figure 1 contains the Manhattan plots for the three phenotypic traits tested in our association analysis across the entire genome (DSM-III-R symptom group counts, DSM-IV symptom group counts, DSM-IV diagnosis with clustering). Significance of the variants was further validated using LD pruning, winsorization of the phenotype values, application of one million permutations, and the inclusion of the covariates age and gender. Although there were 48 total variants which exhibited p-values <= 1E-05, only one variant (rs991316) showed suggestive significance across all three phenotypic categories. This protective variant (rs991316) is located downstream from the alcohol dehydrogenase 7 gene (chr4:100322445 in hg19) and is common in our sample, with an allele frequency of 0.40. The variant retained its significance through permutation and winsorization. Additionally, the variant was not removed during LD pruning with PLINK, indicating that this variant was independent of other markers in the region. The association values are shown in Table 2, and the allele and genotype frequencies can be found in Table 3. The minor allele frequency of rs991316 from the 1000 Genomes project was obtained from the dbSNP website (http://www.ncbi.nlm.nih.gov/SNP/). The minor allele frequencies for the HapMap ethnicities were obtained from the International HapMap Project website (http://www.hapmap.org). Figure 2 contains the Manhattan plot of the association results across the *ADH* gene region only, in order to demonstrate that the SNP near *ADH7* is the most highly significant variant in this region. Power analyses using GWAPower [32] showed that the samples contained 80% power to detect an effect explaining 0.053% of the variance.

**Table 2:** **rs991316 association analysis**

**dbSNP RS ID (rs991316)**	**AlcDepClust**	**AlcDep3**	**AlcDep4**
**Association analysis with no covariates**			
Beta +/− StdError	−0.8029 +/− 0.2065	−0.5853 +/− 0.1376	−0.4533 +/− 0.1097
T-stat	−3.8870	−4.2550	−4.1320
P-value	1.013E-04	2.58E-05	4.34E-05
**Association following winsorization**			
Beta +/− StdError	−0.8029 +/− 0.2065	−0.5566 +/− 0.1288	−0.4345 +/− 0.1023
T-stat	−3.8870	−4.322	−4.248
P-value	1.013E-04	1.93E-05	2.65E-05
**Permutation testing**			
Number of permutations performed	630,000	1,000,000	1,000,000
Empirical adaptive P-value	6.35E-05	3.00E-05	3.80E-05
**Association analysis with covars**			
(Unscaled Beta, P-value)			
Additive	−0.8279, 9.869E-05	−0.5886, 1.571E-05	−0.4543, 3.189E-05
Sex	−0.9037, 6.869E-04	−0.7048, 2.748E-04	−0.5195, 8.147E-04
Age	0.02708, 0.4354	0.06998, 4.863E-03	0.04654, 0.01935
(Scaled Beta, P-value)			
Additive		−0.2035, 1.571E-05	−0.1971, 3.189E-05
Sex		−0.1707, 2.748E-04	−0.1579, 8.147E-04
Age		0.1318, 4.863E-03	0.1100, 0.01935

**Table 3:** **Allele and genotype frequencies for rs991316**

**dbSNP RS ID**	**rs991316**
Minor Allele	C
HapMap MAF: MEX	0.36
HapMap MAF: CEU	0.52
HapMap MAF: AFR	0.52
HapMap MAF: ASIAN	0.04
1000GENOMES_AF	0.3970
**Present Study Sample**	
Overall MAF	0.4
**AlcDepClust Phenotype**	
Affected:	
MAF	0.2568
Genotype counts	5/28/41
HWE pvalue	1
Unaffected:	
MAF	0.4347
Genotype counts	66/174/112
HWE pvalue	1
**AlcDep3 and AlcDep4 Phenotype**	
Genotype counts	71/202/153
Frequency	0.1667/0.4742/0.3592
HWE pvalue	0.7631

The minor allele frequency (MAF) of rs991316 from the 1000 Genomes project (1000GENOMES_AF) was obtained from the dbSNP website (http://www.ncbi.nlm.nih.gov/SNP/). The MAFs for the HapMap ethnicities were obtained from the International HapMap Project website (http://www.hapmap.org). Genotypes for the present study are listed in the order: homozygous_minor/heterozygous/homozygous_major. Abbreviations: MAF = minor allele frequency; HWE = Hardy-Weinberg equilibrium; HapMap MAFs: MEX = Mexican ancestry in Los Angeles, California; CEU = Utah residents with Northern and Western European ancestry; AFR = average MAF of ASW (African ancestry in Southwest USA), LWK (Luhya in Webuye, Kenya), MKK (Maasai in Kinyawa, Kenya), and YRI (Yoruban in Ibadan, Nigeria); ASIAN = average MAF of CHB (Han Chinese in Beijing, China), CHD (Chinese in Metropolitan Denver, Colorado), and JPT (Japanese in Tokyo, Japan).

### Multiple test correction

Multiple test correction p-value thresholds were calculated for the Affymetrix Exome1A chip using the Genetic Type 1 Error Calculator (GEC) software [25], and the thresholds generated were thereby used to determine that this variant (rs991316) could be characterized to possess suggestive significance.

### LD analysis

**Figure 3:**
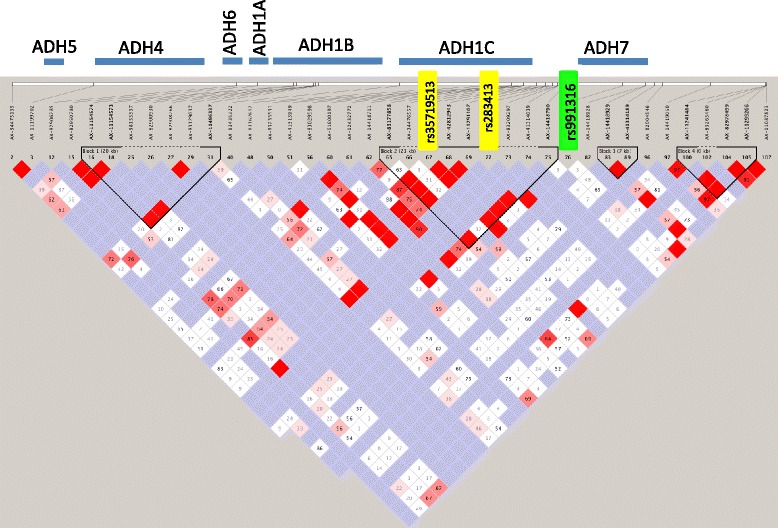
**Linkage disequilibrium (LD) plot across ADH gene cluster.** This plot illustrates the linkage disequilibrium between SNPs genotyped across chromosome 4 encompassing the *ADH* gene cluster. LD was measured using D’. Blue bars above the plot indicate the gene locations. Increasing levels of LD are indicated by pink and red squares, with red squares demonstrating the highest confidence linkage estimates. The green rectangle highlights SNP rs991316. The yellow rectangles highlight the two SNPs in ADH1C that were in LD with SNP rs991316.

The evidence of LD across the *ADH* gene region can be observed in Figure 3. The average D’ value across the SNP pairs in the *ADH* region was 0.76 in this data set. The two SNPs found to be in complete LD with *ADH7* (rs991316) with high confidence were: rs283413 (D’ = 1, r^2^ = 0.025, LOD = 3.48, CI = 0.56-1.00), and rs35719513 (D’ = 1, r^2^ = 0.03, LOD = 2.3, CI = 0.41-1.00). Both of these SNPs were located in *ADH1C* (rs283413, chr4:100268190; and rs35719513, chr4:100260783) and possessed a negative beta value, suggesting a protective role. However, these SNPs were not significantly associated with the alcohol dependence phenotypes in our sample when using PLINK for the analysis as can be seen in Figure 2.

### Collapsing/Gene-based analysis

The three collapsing techniques tested did not produce any significant p-values for the *ADH* gene region (p-value = 1.0 in all cases). Based on these results, we did not further pursue any other collapsing techniques for this data set.

## Discussion

### Demographics

The prevalence of alcohol dependence in this cohort ranged from 17% to 29%, depending upon the criteria used for the diagnosis, as shown in Table 1. DSM-IV alcohol dependence where the symptoms clustered within a 1-year period had the lowest prevalence rate at 17% overall, with a rate of 25% in males and 12% in females. The alcohol dependence diagnosis based on DSM-III-R had the highest overall prevalence of 29%, with 36% in males and 24% in females. The alcohol dependence diagnosis based on DSM-IV without requiring clustering was between these two ranges, at an overall rate of 20%, with a rate of 28% in males and 15% in females. Our values agree with the previous reports that males have a higher rate of alcohol dependence than females [33]. However, these values are higher than the reported rate of 9.5% for lifetime alcohol dependence in Hispanics across all age groups [34]. Since overall alcohol dependence prevalence in the U.S. is highest in the 18–30 year old age group [7,34,35], this may have contributed to the larger values in our study.

Recent studies have also examined the role of acculturation stress on Mexican Americans [35-37]. In one study, alcohol dependence in the past 12-month period was compared in Mexican Americans that lived in counties on the U.S.-Mexico border versus counties that are not near the border [35]. In that study, the alcohol dependence rate based on DSM-IV for 18–29 year old males was 24.0% for those living in bordering counties and 18.8% for those not in bordering counties. Since our subjects live in a border county, they may be exhibiting this higher rate based upon their location. In the same study, the prevalence for females also followed this same trend of higher rates in border counties, but at a lower rate than males. In particular, the rates for 18–29 year old females was 6.4% in border counties, and 1.7% in non-border counties. These reported lower values than those found in our sample, and may be due to the difference between past 12-month rates as examined in that study and lifetime rates of alcohol dependence as examined in the present study.

### Association analysis

The results of the present study suggest that a variant (rs991316) located downstream of the alcohol dehydrogenase 7 gene (*ADH7*) may play a role in protection from alcohol dependence in this Mexican American cohort. Variants in the alcohol dehydrogenase (*ADH*) and aldehyde dehydrogenase (*ALDH*) genes have been found to influence alcohol metabolism, and thereby contribute to the effect that alcohol exerts on an individual. *ADH7*, in particular, is expressed in the stomach mucosa to metabolize alcohol, and therefore, provides the first line of defense against the effects of alcohol before it enters the blood [38]. The *ADH7* gene has been shown to have a protective role against alcohol dependence in previous studies involving other ethnicities, but to our knowledge, has not been reported for Mexican Americans [39-41]. Additionally, this *ADH7* SNP (rs991316) has not been identified to be significantly associated with alcohol dependence. Instead, this SNP was deposited into the dbSNP database (http://www.ncbi.nlm.nih.gov/SNP/) based on a study of hypertension in African Americans [42].

Recently, our group has reported a significant association of rs991316 with alcohol dependence in a Native American (NA) cohort [43]. In particular, in a whole genome sequencing study of Native Americans, rs991319 had a MAF of 0.347, a significant FDR p-value (0.0458), and a negative beta, suggesting a protective role. The NA sample (n = 320) had a higher rate of alcohol dependence of 46%, while the MA cohort had an alcohol dependence of approximately 20%. In addition to the whole genome sequencing data, Affymetrix Exome1A array data was available for the NA cohort. The MAF was 0.376 for the rs991316 marker for the NA cohort with the Affymetrix data, which validated the MAF obtained by the whole genome sequencing. Because variants located in the *ADH* gene region have been found to be associated with alcohol dependence in a Native American population [18,40,44], we also examined the self-reported ancestry for this Mexican American cohort. Of the 427 participants in the cohort, only 44 reported any amount of Native American heritage, which did not provide power for admixture analysis.

### LD analysis

Because this particular variant near *ADH7* (rs991316) had not been previously reported to be associated with alcohol dependence, we wanted to rule out the possibility that this effect was simply the result of LD with a variant in one of the other *ADH* genes. The LD analysis produced a high average LD across the entire *ADH* gene region (D’ = 0.76), demonstrating that this region is important in alcohol dependence. However, as can be seen in Figure 3, there are very few individual SNPs that are in complete LD with rs991316. This result was expected based on previous reports that there is not strong LD between *ADH7* and the other *ADH* gene classes [39]. Even within the *ADH7* gene itself, there exists substantial diversity in the haplotypes in a 23 kb region around *ADH7*, within and among 38 global populations [45].

Two variants in *ADH1C*, rs238413 and rs35719513, were found to be in complete LD with rs991316 in this study. These two SNPs had a negative beta, suggesting a protective role, but were not significantly associated with the alcohol dependence phenotypes used in this study. These two variants have been studied previously to determine their role in the rate of alcohol metabolism in white Spanish individuals [46]. In that study, rs283413 was significantly associated with the rate of alcohol metabolism, but rs35719513 was not.

The *ADH1C* gene has been associated with alcohol dependence in many previous studies involving various ethnic groups, including Mexican Americans [15,17], European Americans [47,48], African Americans [47,48], Native Americans [44], Asians [49], Israelis [50], North Africans from Morocco and Southern Europeans from Basque Country [51] and Afro-Caribbeans [52]. These studies, along with a meta-analysis comprised of 53 previous reports [49], also demonstrate the differences in allele frequencies of variants in *ADH1C* across the global populations.

Several genotyping studies have targeted alcohol dependence in Mexican Americans, focusing on the genes involved in alcohol metabolism (*ADH*, *ALDH*, and *CYP2E1*), and a subset of neurotransmitter-related genes (*DRD2*, *5-HTTLPR*, and *GABRβ3*) [14-18]. These studies found a significant association to variants in *ADH1B*, *ADH1C, CYP2E1 RasI c2* allele, *DRD2 -141C* insertion/deletion, and *5-HTTLPR S* allele. These previous studies did not find a significant association with *ALDH* or *GABRβ3*. The Affymetrix Axiom Exome 1A array does not contain markers for CYP2E1*5B, DRD2-141C, or the *5-HTTLPRS S* allele, so we were not able to detect associations for these variants. It may also be the case that these previous studies were candidate gene approaches and did not use exome-wide correction as we have performed in this study. Additionally, we may have missed some associations due to low sample size and loss of power. However, the rs991316 finding is supported given that this SNP showed an association with alcohol dependence in this cohort and in an independent sample of Native Americans.

## Conclusions

A number of factors have been found to contribute to alcohol dependence in Mexican Americans. Recent studies have found Mexican Americans living in counties bordering the U.S. to be particularly prone to high rates of alcohol dependence [35]. The current study is the first exome-wide association to be reported for a cohort of Mexican Americans living in a border region of the United States. By performing an exome-wide association with the Affymetrix Exome1A array using both dichotomous and quantitative phenotypes for alcohol dependence in Mexican Americans, this study was able to examine genes in the *ADH* region, as well as other genes previously implicated in alcohol dependence with other ethnicities. The *ADH7* gene region contained the most significant variant in the three phenotypes that we evaluated. This result is in agreement with previous studies that *ADH7* has a role in the protection with alcohol dependence [39], although it was not tested previously in Mexican Americans. Therefore, this study contributes to the emerging picture of genetic variation in alcohol dependence in Mexican Americans.
